# The effect of auricular vagus nerve stimulation on electroencephalography and electromyography measurements in healthy persons

**DOI:** 10.3389/fphys.2023.1215757

**Published:** 2023-07-17

**Authors:** Gülşah Konakoğlu, Ali Veysel Özden, Hakan Solmaz, Celaleddin Bildik

**Affiliations:** ^1^ Faculty of Health Sciences, Istanbul Gelisim University, Istanbul, Türkiye; ^2^ Faculty of Health Sciences, Bahçeşehir University, Istanbul, Türkiye; ^3^ Faculty of Engineering and Natural Sciences Biomedical Engineering Department, Bahçeşehir University, Istanbul, Türkiye; ^4^ Ataşehir Florence Nightingale Hospital, Istanbul, Türkiye

**Keywords:** auricular vagus nerve stimulation, surface electromyography, electroencephalography, muscle activation, frequency band power

## Abstract

**Objectives:** Auricular vagus nerve stimulation (VNS) is a non-invasive treatment modality. Opinions that it can be used in the treatment of various clinical problems have gained importance in recent years. In this study, it was aimed to lay the groundwork for the use of the auricular VNS in different ears.

**Methods:** Healthy individuals (*n* = 90) were divided into three groups: unilateral left (*n* = 30), unilateral right (*n* = 30), and bilateral (*n* = 30) auricular VNS. Electroencephalography (EEG) and electromyography (EMG) measurements were performed before and after auricular VNS (10 Hz, 300 µs, 20 min) for a single session.

**Results:** An increase in wrist extensor muscles activation was detected on the contralateral side of the auricular VNS application side. It has been observed that there is a general decrease in the power of high-frequency waves and an increase in the power of lower-medium frequency waves in various parts of the brain.

**Conclusion:** Our findings suggest that the projection of the auricular VNS in the central nervous system may also affect the corticospinal tracts.

## 1 Introduction

The autonomic nervous system (ANS) is the part that regulates a large part of the body’s visceral functions (Snell, 2010). Parasympathetic and sympathetic divisions of the ANS, containing both afferent and efferent nerve fibers, are distributed throughout the central nervous system (CNS) and peripheral nervous system (Guyton and Hall, 1986). Efferent autonomous signals are transmitted to various organs of the body by these two basic parts. Approximately 75% of all parasympathetic fibers travel within the vagus nerve (10th cranial nerve). Therefore, when the parasympathetic system is mentioned, the first vagus nerve comes to mind (Gökçe et al., 2018). It is reported that the vagus nerve plays a role in homeostasis, stress control and regulation of the immune system. The vagus nerve, which has nuclei in the brain stem, descends from the neck and connects with almost all internal organs (Ohemeng and Parham, 2020). Since the shape or placement of visceral organs is also not symmetrical, the neural network of the ANS requires asymmetry and central regulation is lateralized through the vagus. The neural control of the vagus is ipsilateral. For example, the left vagus nerve originates from the left side of the brain stem. The nuclei of the vagus nerve in the brain stem are the nucleus of the solitary tract (NTS, afferent), nucleus spinalis of the trigeminal nerve (NSNT, afferent), nucleus ambiguous (NA, efferent), and dorsal motor nucleus (DMN, efferent). The fibers on both sides, the left and right branches of the vagus nerve, originate ipsilaterally from nuclei in the brain stem, either from the DMN or NA. Both the DMN and NA are also lateralized (Porges et al., 1994).

VNS defines any technique that stimulates the vagus nerve. It can be performed from the cervical region with invasive methods, as well as from the cervical or auricular region with non-invasive methods. Electrical stimulation is the main form of stimulation. In addition to its wide distribution in the body, VNS can be used in many different indications thanks to the connections of the nuclei in the brain stem (Farmer et al., 2016).

Auricular VNS is simpler and cheaper compared to invasive methods. There are also no complications caused by surgery and side effects as a result of stimulation of efferent fibers. Auricular VNS stimulates only afferent fibers and provides additional advantages such as the bilateral application. For all these reasons, auricular VNS as a non-invasive method comes to the fore due to its necessity in treatment (Özden et al., 2021). Although it is an easily administered treatment with few side effects, it remains a problem to find the appropriate stimulation parameters for different clinical problems and to make a clinical decision about which ear should be stimulated.

The effect of auricular VNS on the interaction between the ANS and the CNS can be determined by different measurement methods such as functional magnetic resonance imaging (fMRI), electroencephalography (EEG), electrocardiography (ECG), microneurography and pupillometry (Fallgatter et al., 2003; Kraus et al., 2013; Leutzow et al., 2013; Clancy et al., 2014; Hagen et al., 2014; Frangos et al., 2015; Antonino et al., 2017; Yakunina et al., 2017; Badran et al., 2018; Tu et al., 2018; Sclocco et al., 2019; Warren et al., 2019). Machetanz et al. (2021) reported that trigeminal and sympathetic circuits should also be taken into account by using additional measurement methods such as electromyography (EMG) in future studies, and more detailed comparisons should be made between right and left auricular stimulation, as a result of their research on the brain-heart interaction with simultaneous EEG and ECG measurements.

It has been shown that the auricular VNS has effects on cerebral activity (Silvani et al., 2016; Kibleur et al., 2018; Patron et al., 2019; Keute et al., 2021; Machetanz et al., 2021). Therefore, in this study, the effect of VNS on brain frequency bands was evaluated with by means of EEG measurements. Maximum isometric grip strength was measured simultaneously using a hand dynamometer, which was connected to the same equipment during the activation measurements of the wrist extensor muscle with a surface EMG device. Thus, the peripheral effect of auricular VNS has been investigated.

The aim of this study was to evaluate the cortical and peripheral effects of VNS applied from different ears. The participants were divided into three groups as “*right unilateral*”, “*left unilateral*” and “*bilateral” auricular VNS*. The participants, who were grouped to be stimulated from different ears, were evaluated in terms of EEG and EMG measurements performed before and after stimulation.

## 2 Materials and methods

### 2.1 Participants

In this study, healthy individuals of ages of between 18 and 45, who did not have any chronic diseases and do not use any medication regularly, were included in the three experimental groups. The volunteers included in the study did not also have any surgery of upper extremities (shoulder, elbow, wrist and fingers). Individuals with orthopedic problems were excluded from the study in order to prevent any inaccurate of the superficial EMG measurements taken from the upper extremities. In addition, individuals with any systemic disorder history such as diabetes, gout, chronic kidney failure, rheumatoid arthritis, thyroid diseases were also excluded from the study.

Participants were reached through e-mail groups and social media announcements in the institutions we are affiliated with. Healthy individuals who accepted to participate in the study as voluntary participants and met the inclusion criteria were included in the study on a voluntary basis. The volunteers were randomly assigned to three groups to perform right-unilateral (21 female, 9 male; 20–44 years old), left-unilateral (16 female, 14 male; 21–44 years old) and bilateral (23 female, 7 male; 22–45 years old) auricular VNS. A total of 90 participants (60 female, 30 male; 20–45 years old), including 30 healthy subjects in each group.

### 2.2 Procedure

The study was approved by a local ethical review board (T.R. Istanbul Yeni Yüzyıl University Clinical Research Ethics Committee, Approval number: 08.04.2021/18) and registered to Clinical Trials (ClinicalTrials.gov number, NCT05088135).

In the first stage, the participants were informed about the research, and were asked to sign a written consent. Demographic information and, Body Mass Index (BMI) of the volunteers were recorded. The subjects were asked not to smoke and caffeine for at least 2 h, and also not to drink alcohol for the last 24 h before the auricular VNS. Heavy work or exercise of upper extremities was also forbidden before the measurements.

In the literature, it has been reported that the wrist extensor muscle activation is higher than the wrist flexor muscle activation during maximum isometric grip (Konakoğlu, 2018). For this reason, before and after the auricular VNS, activation of the wrist extensor muscle groups was measured simultaneously by superficial EMG in both wrist grip strength measurements using a hand dynamometer. In addition, EEG measurement was performed to examine the effect of stimulation on the band power of brain frequencies. These EEG frequency bands are theta, alpha, low beta, high beta and gamma.

**FIGURE 1:**
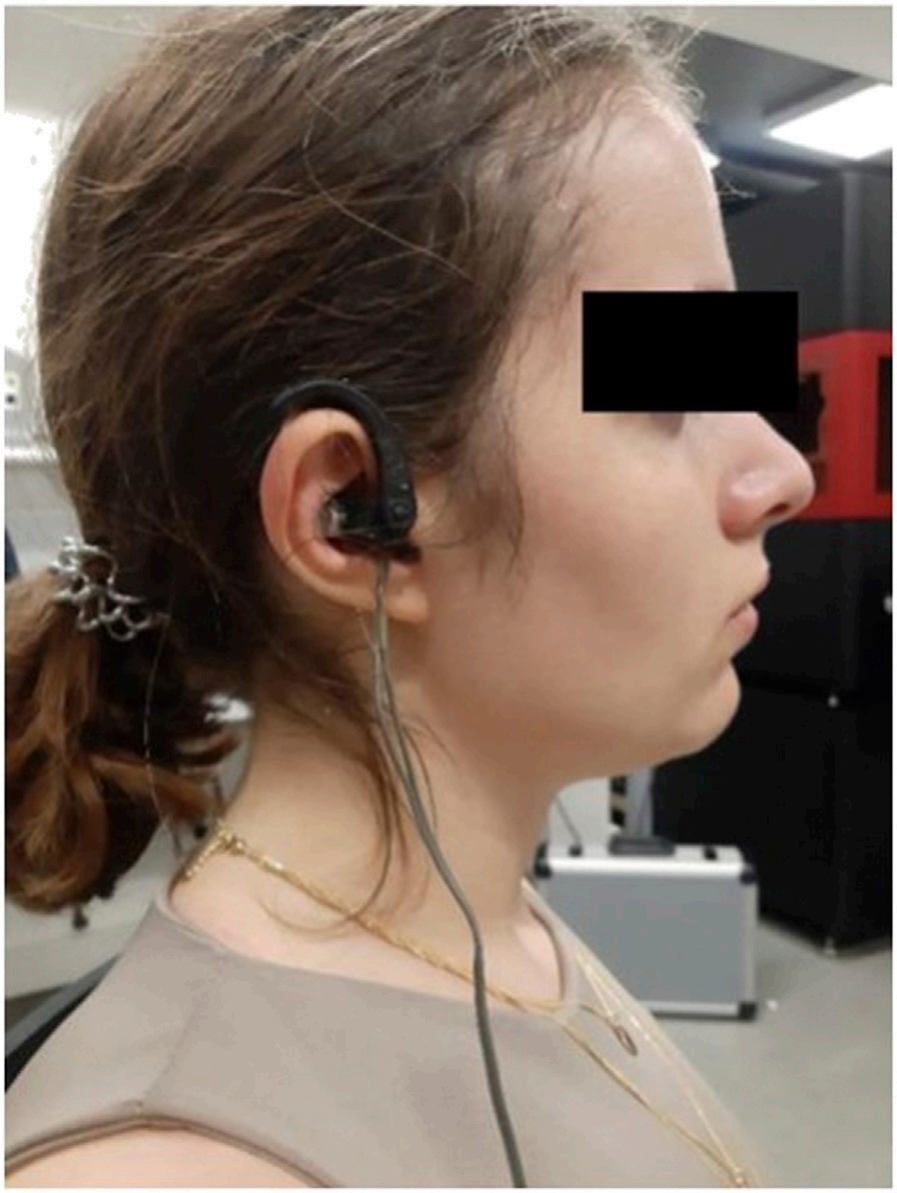
Example of right auricular VNS electrode placement.

Auricular VNS between the first measurement and the last measurement was performed by the auricular nerve stimulation device Vagustim (Vagustim Health Technologies, Teknokent, Istanbul, Türkiye). Auricular VNS was performed from tragus and concha regions, for 20 min with a frequency of 10 Hz and a pulse width of 300 µs in all three groups (Figure 1). The difference in stimulation between the groups depends only on to which ear it was applied.

Healthy volunteers were divided into three groups to perform “right unilateral”, “left unilateral” and “bilateral” auricular VNS.

### 2.3 Electrophysiological data recording and processing

#### 2.3.1 EMG

Measurement of wrist extensor muscle activation *(mV)* was performed on the healthy volunteers with the BIOPAC (Biopac Systems, Santa Barbara, CA, United States) EMG device and the measurement of hand grip strength was done with a hand dynamometer connected to the same device was simultaneously measured. Measurements were performed on both wrist extensor muscles before and after the auricular VNS.

The measurements of the wrist extensor muscle were done using three Ag/AgCl EMG surface electrodes. The grounding electrode was placed on the ulnar styloid process. The other two surface electrodes were placed on the wrist extensor muscle at approximately 0.5 cm intervals. Simultaneously with the surface EMG measurement of wrist extensor group muscle activation, maximum isometric hand grip strength was measured in terms with of clench force *(kg)* the hand dynamometer of the same device.

**FIGURE 2:**
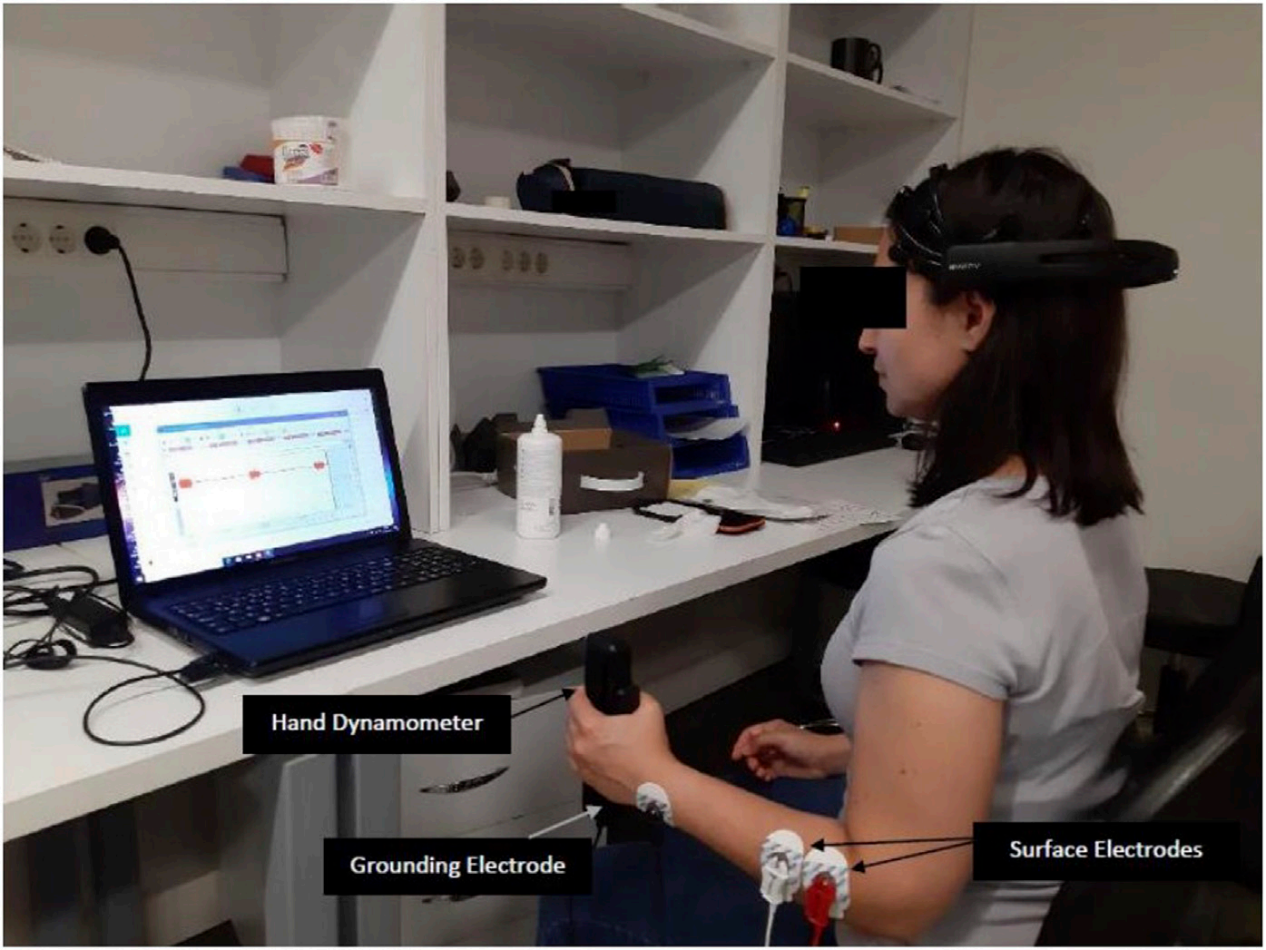
Measurement position and placement of electrodes.

**FIGURE 3:**
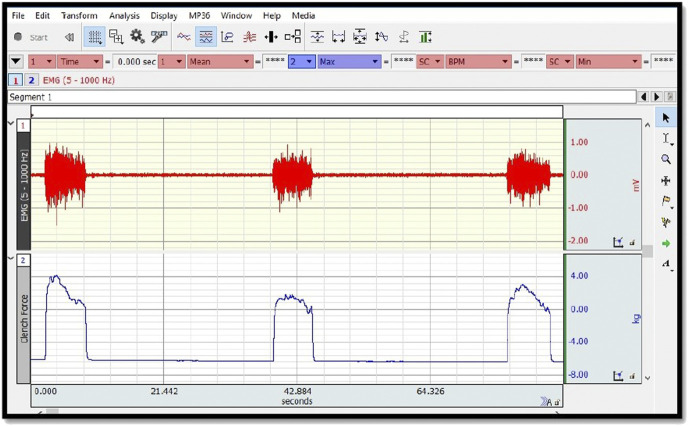
EMG measurement graph.

The measurements were held in the standard measurement position of the hand dynamometer, with 0° shoulder flexion, 0° shoulder abduction, 90° elbow flexion, and with the forearm in the midline position in the sitting position, during 5 s of maximum grip (Figure 2). The measurements were triplicated with 30 s of resting interval. The root-mean-square (RMS) average of measurement results for each subject was calculated in MATLAB software (Figure 3). The average of three measurements made for both parameters was calculated. The measurement was repeated both wrists. EMG measurements were performed on both wrist extensor muscles before and after the auricular VNS.

#### 2.3.2 EEG

**FIGURE 4:**
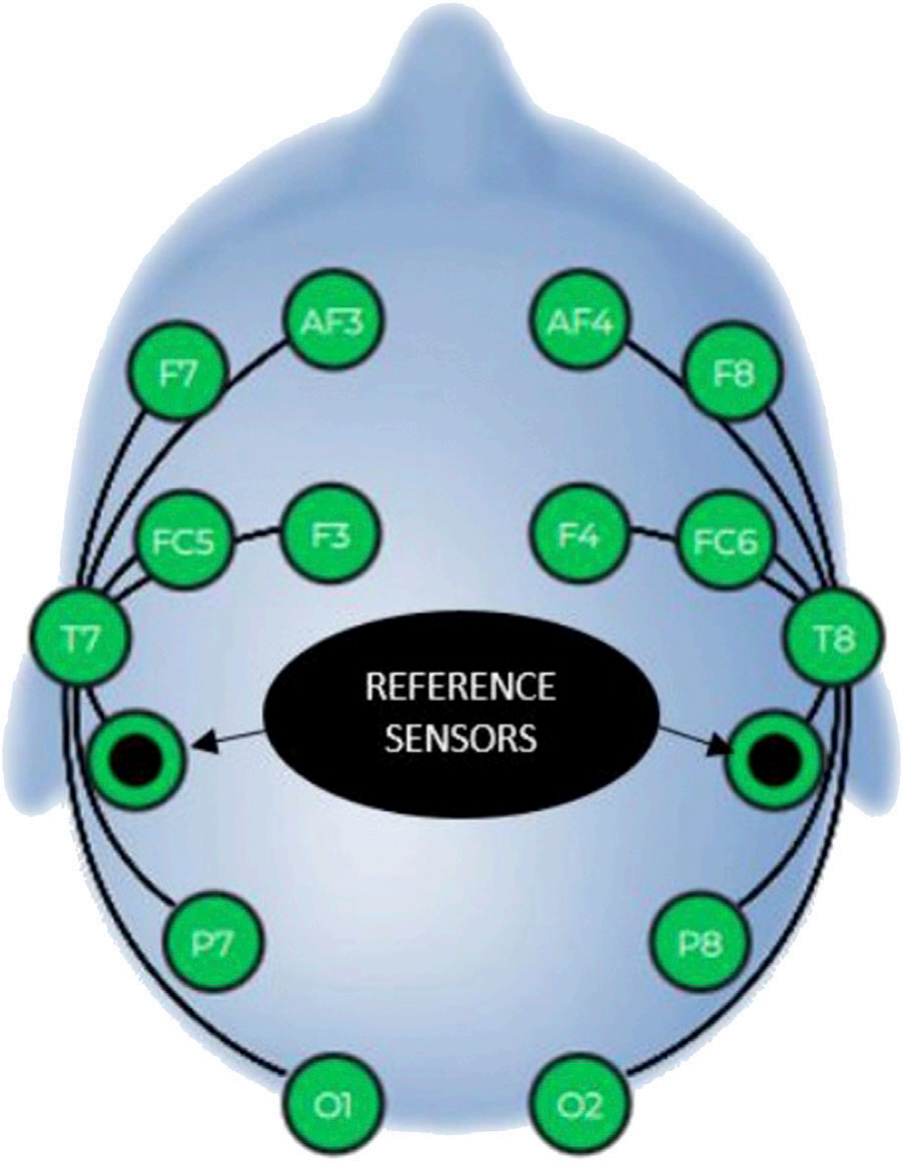
Placement of reference and electrode sensors.

The band power of the frequencies of brain waves were measured with 14-channel mobile Emotiv EPOC + (EMOTIV Inc., San Francisco, CA, United States) EEG device for 10 min before and after the auricular VNS. According to the layout specified in the manual of the device, starting from the top of the skull, reference electrodes were positioned in contact with the skull just behind each earlobe. Both frontal electrodes on the right and left sides were approximately three fingers above the eyebrows. The Emotiv EPOC + EEG device uses the international 10–20 system. It includes the frontal and prefrontal lobes, as well as the temporal, parietal, and occipital lobes. The electrode sensors used were AF3, F7, F3, FC5, T7, P7, O1, O2, P8, T8, FC6, F4, F8, AF4 (Figure 4).

**FIGURE 5:**
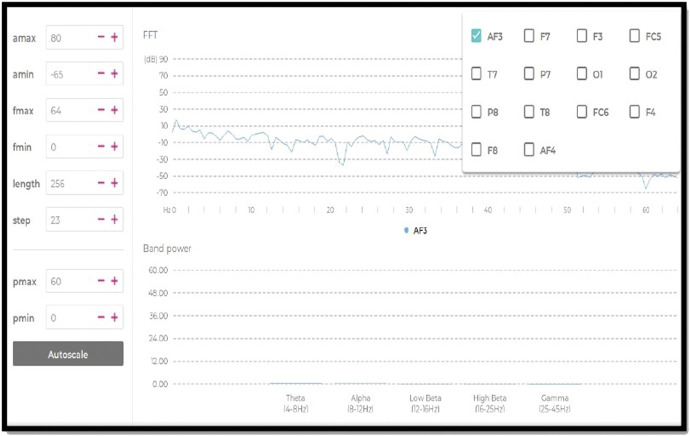
Graph of band power during the placement.

Five separate frequency band power values were provided for each electrode (Figure 5). These frequency bands are theta (4–8 Hz), alpha (8–12 Hz), low beta (12–16 Hz), high beta (16–25 Hz) and gamma (25–45 Hz). The contact quality of each electrode was tested to achieve a full contact of the electrodes with the skin surface before starting the measurements.

EEG measurements were performed before and after the stimulation for 10 min with the eyes of the participants closed. Factors such as sound and light that may distract the participants were blocked and it was checked whether each participant was awake.

The data obtained from the measurement were saved in CSV (Comma Separated Values) format, and then the “average band power” was analyzed in Excel. The band power (μV2) of the frequencies of each of the 14 channels was calculated. The obtained EEG data were analyzed by dividing the brain into four quadrants. Accordingly, “AF3 + F7 + F3 + FC5” formed the left anterior region, “AF4 + F8 + F4 + FC6” formed the right anterior region, “T7 + P7 + O1” formed the left posterior region, and “T8 + P8 + O2” formed the right posterior region. The average band power (μV^2^) of theta, alpha, low beta, high beta, and gamma frequencies of each of these regions was calculated.

### 2.4 Statistical analysis

SPSS 22.0 Package Program was used for statistical analysis. Descriptive statistics of the variables used in the study, number (n), and frequency values were used in categorical data. In continuous data, mean, standard deviation, and minimum maximum values were used. First of all, it was examined whether the continuous variables had a normal distribution according to the Shapiro-Wilk test. Non-parametric tests were used for non-normally distributed variables (*p* < 0.05), and parametric tests were used for normally distributed variables (*p* > 0.05). To measure the difference between the means of the two dependent groups; Paired-Sample *t*-Test for normally distributed data and Wilcoxon Sign Rank Test for non-normally distributed data were used. The significance level was given as 0.05 in all analyzes.

## 3 Results

### 3.1 Demographic findings

There was no significant difference in gender distribution between the groups (*p* = 0.142). Similarly, no significant difference was found between the groups in age (*p* = 0,13), and BMI measurements (*p* = 0.067). Findings regarding age and BMI measurements are shown in Table 1.

**TABLE 1:** Baseline characteristics. There was no significant difference in baseline characteristics between left-unilateral, right-unilateral and bilateral auricular VNS groups.

Auricular VNS Group		Mean ± SD	Min-Max
Left-Unilateral (n = 30)	Age (years)	31.5 ± 6.65	21–44
	BMI (kg/m^2^)	23.64 ± 4.36	16.23–37.46
Right-Unilateral (n = 30)	Age (years)	28.23 ± 6.078	20–44
	BMI (kg/m^2^)	24.16 ± 4.58	18.72–37.88
Bilateral (n = 30)	Age (years)	31.17 ± 8.192	22–45
	BMI (kg/m^2^)	21.79 ± 3.24	16.14–31.56
Total (n = 90)	Age (years)	30.30 ± 7.107	20–45
	BMI (kg/m^2^)	23.19 ± 4.18	16.14–37.88

### 3.2 EMG data findings

In the group in which we performed left auricular VNS, the mean activation value (mV) of the right wrist extensor group muscles obtained after stimulation was found to be significantly higher than the value before stimulation (*p* = 0.038). There was no significant difference in the mean activation value of the left wrist extensor group muscles after stimulation in the same group compared to before stimulation (*p* > 0.05). In the right auricular VNS group, the mean activation value (mV) of the left wrist extensor group muscles obtained after stimulation was found to be significantly higher than the value before stimulation (*p* = 0.021). In this group, no significant difference was found in the mean activation value of the right wrist extensor group muscles after stimulation compared to before stimulation (*p* > 0.05). The mean activation value (mV) of both the left and right wrist extensor group muscles after stimulation was found to be significantly higher than the pre-stimulation value in the group in which we performed bilateral auricular VNS (*p* < 0.05). In the measurements, no significant difference was found in the maximum isometric hand grip strength (kg) of both the right and left sides after auricular VNS in all three groups compared to the pre-stimulation (*p* > 0.05). The findings regarding the maximum hand grip strength and wrist extensor group muscle activation measurements before and after VNS in the groups participating in the study are shown in Table 2.

**TABLE 2:** Hand grip strength and wrist extensor muscle activation.

		Before VNS		After VNS		Z-t^b^	*p*
		Mean ± SD	Min-Max	Mean ± SD	Min-Max		
Left —Unilateral (*n* = 30)	Right Wrist Extensor Muscle Activation (mV)	0,268 ± 0,103	0,122–0,472	0,288 ± 0,111	0,122–0,479	−2,172	**0,038^b^**
	Right Hand Grip Strength (kg)	14,747 ± 9,111	3,551–40,207	14,878 ± 9,655	3,01–38,079	−0,134	0,894^a^
	Left Wrist Extensor Muscle Activation (mV)	0,227 ± 0,087	0,060–0,504	0,247 ± 0,108	0,113–0,633	−1,254	0,252^a^
	Left Hand Grip Strength (kg)	13,543 ± 8,217	2,388–32,208	14,303 ± 9,329	2,342–32,702	−1,399	0,162^a^
Right - Unilateral (n = 30)	Right Wrist Extensor Muscle Activation (mV)	0,261 ± 0,117	0,069–0,596	0,278 ± 0,129	0,108–0,695	−1,72	0,085^a^
	Right Hand Grip Strength (kg)	13,269 ± 8,061	4,394–34,819	13,205 ± 7,783	3,115–36,731	−0,113	0,91^a^
	Left Wrist Extensor Muscle Activation (mV)	0,290 ± 0,213	0,091–1,238	0,319 ± 0,268	0,086–1,578	−2,304	**0,021^a^**
	Left Hand Grip Strength (kg)	12,343 ± 7,723	3,034–31,877	12,406 ± 7,908	1,086–34,551	−0,616	0,538^a^
Bilateral (*n* = 30)	Right Wrist Extensor Muscle Activation (mV)	0,295 ± 0,157	0,101–0,841	0,318 ± 0,173	0,095–0,787	−2,819	**0,005** ^a^
	Right Hand Grip Strength (kg)	10,978 ± 6,947	3,191–29,232	11,136 ± 6,948	1,406–31,111	−0,483	0,629^a^
	Left Wrist Extensor Muscle Activation (mV)	0,265 ± 0,119	0,117–0,541	0,284 ± 0,127	0,107–0,523	−1,985	**0,047** ^a^
	Left Hand Grip Strength (kg)	9,693 ± 5,990	2,725–25,035	9,718 ± 6,150	3,144–28,075	−0,195	0,845^a^

^a^ Wilcoxan Sign Rank Test.

^b^ Paired-Sample *t*-Test.

The bold values are *p* < = 0.

### 3.3 EEG data findings

**FIGURE 6:**
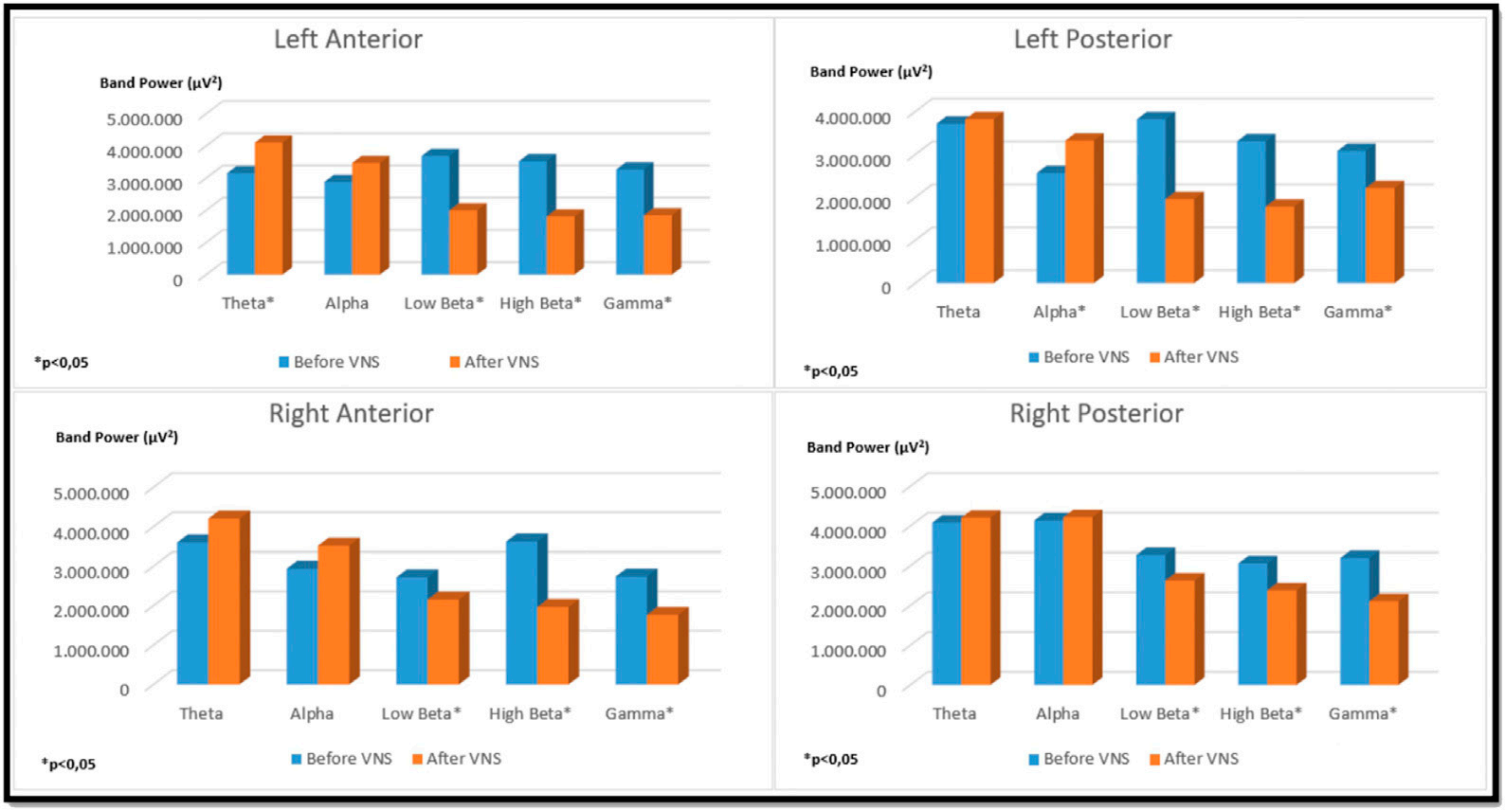
Comparison of religional band power before and after simultation in the left-unilateral auricular VNS group.

**FIGURE 7:**
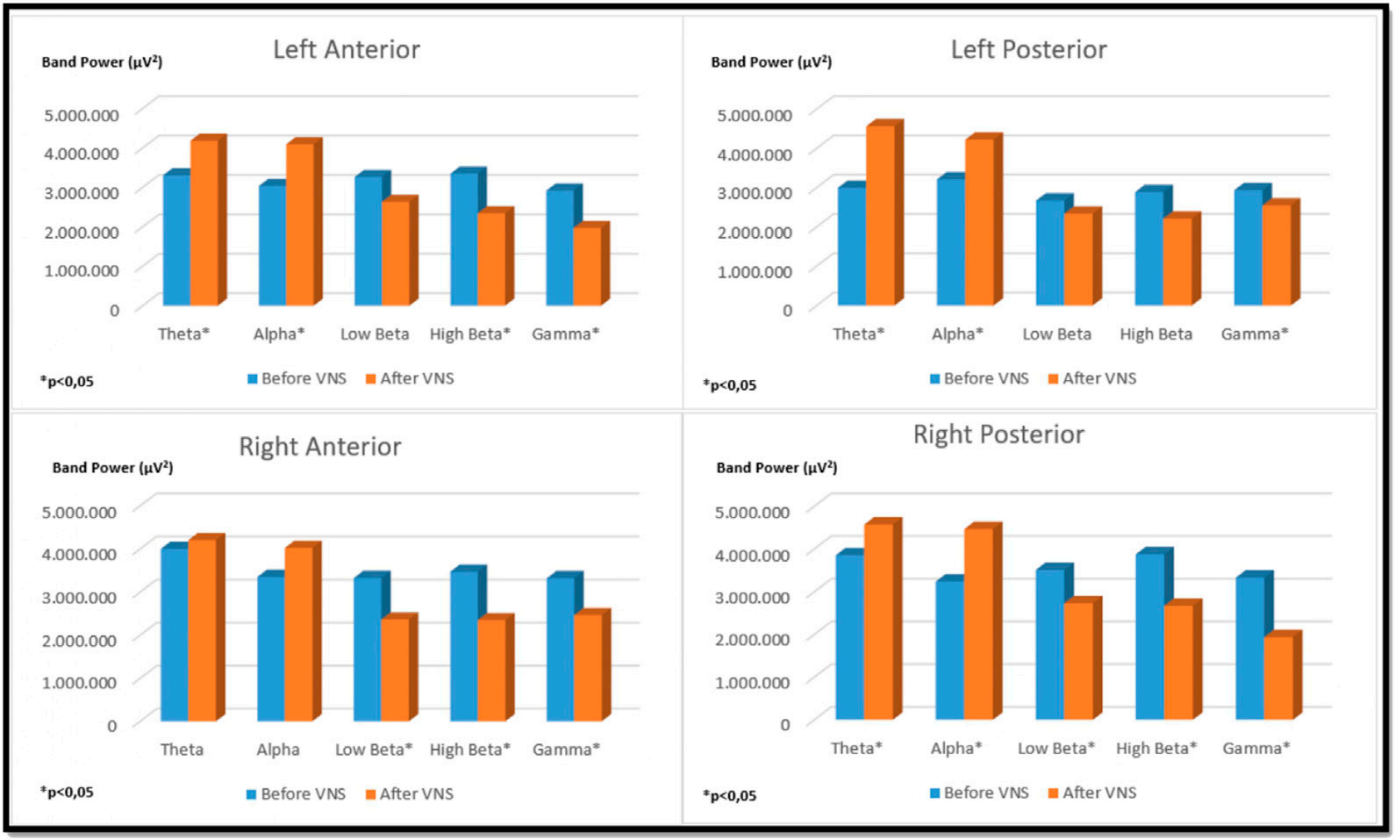
Comparison of religional band power before and after simultation in the right-unilateral auricular VNS group.

In the left auricular VNS group, a significant increase was found in theta frequency band power in the left anterior region and in the alpha frequency band power in the left posterior region compared to the pre-stimulation (*p* < 0.05). When the low beta, high beta and gamma frequency band power were examined in the four regions of the brain in the same group, it was determined that there was a significant decrease compared to the pre-excitation (*p* < 0.05) (Figure 6). In the right auricular VNS group, there was a significant increase in theta and alpha frequency band power in the left anterior, left posterior and right posterior regions compared to pre-stimulation (*p* < 0.05). In the same group, when the change in low beta frequency band power was compared with the pre-stimulation, there was a significant decrease in the right anterior and right posterior regions (*p* < 0.05). Considering the change in high beta frequency band power, it is seen that there is a significant decrease in the left anterior, right anterior and right posterior regions compared to pre-stimulation (*p* < 0.05). It was determined that there was a significant decrease in gamma frequency band power in all regions compared to pre-excitation (*p* < 0.05) (Figure 7).

**FIGURE 8:**
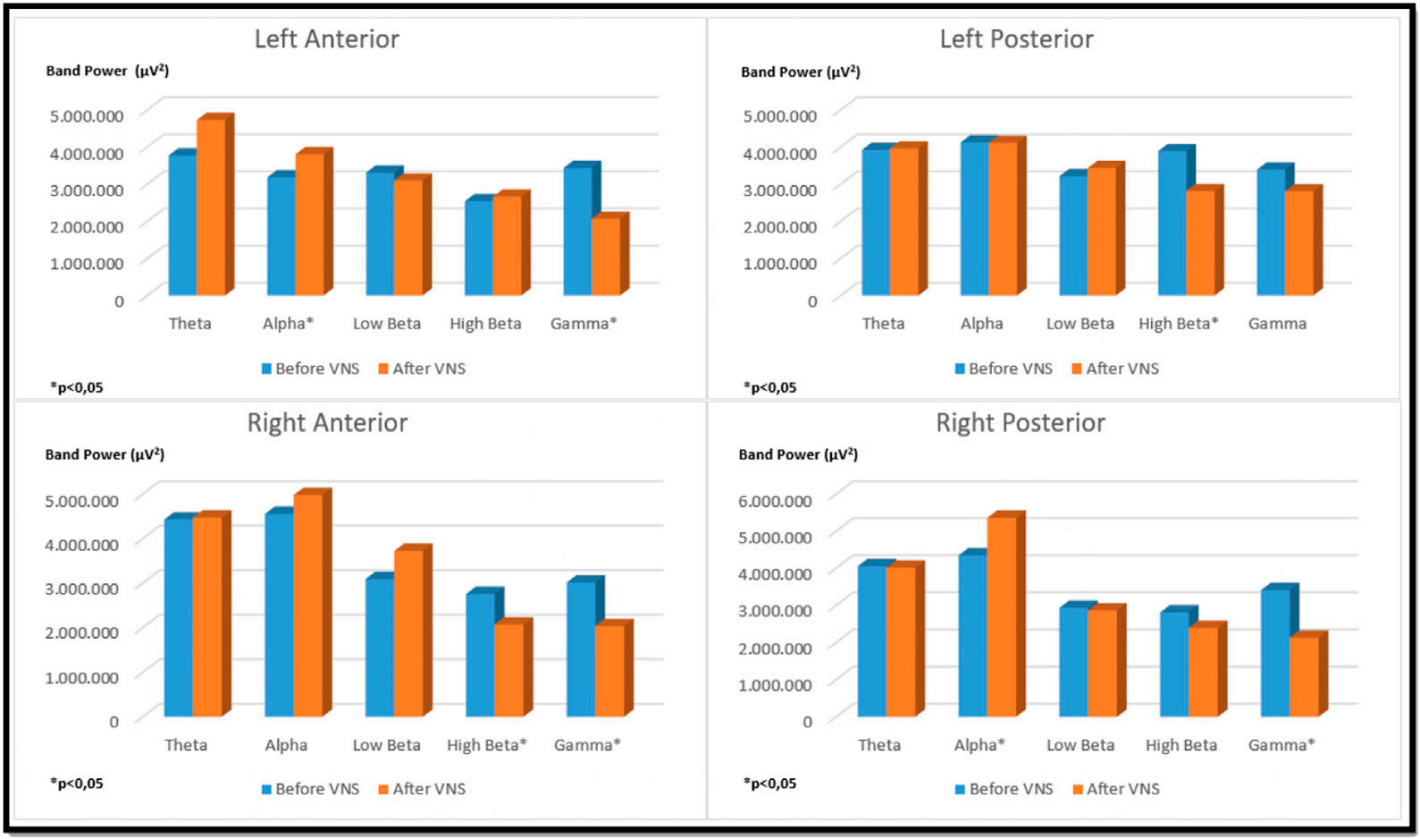
Comparison of religional band power before and after simultation in the bilateral auricular VNS group.

There was a significant increase in alpha band strength in the left anterior and right posterior regions in the bilateral auricular VNS group compared to pre-stimulation (*p* < 0.05). When the change in high beta frequency band power in the same group was compared with the pre-stimulation, it was observed that there was a significant decrease in the right anterior and left posterior regions (*p* < 0.05). Considering the change in gamma frequency band power, it was seen that there is a significant decrease in the left anterior, right anterior and right posterior regions compared to pre-stimulation (*p* < 0.05) (Figure 8). The findings regarding the power of theta, alpha, low beta, high beta and gamma frequency bands measured by EEG before and after VNS in the groups participating in the study are shown in Table 3.

**TABLE 3:** Comparison of regional band powers before and after stimulation in the left-unilateral, right unilateral and bilateral auricular VNS groups.

		Region	Before VNS		After VNS		Z-t^b^	*p*
			Band power (μV^2^)		Band power (μV^2^)			
			Mean ± SD	Min-Max	Mean ± SD	Min-Max		
THETA	Left Unilateral	Left Anterior	3,15 × 10^6^ ± 1,39 × 10^6^	1,04 × 10^6^–7,21 × 10^6^	4,1 × 10^6^ ± 1,87 × 10^6^	1,71 × 10^6^–9,81 × 10^6^	−2,396	**0,017^a^**
		Left Posterior	3,72 × 10^6^ ± 2,02 × 10^6^	1,43 × 10^6^–9,9 × 10^6^	3,82 × 10^6^ ± 2,09 × 10^6^	1,24 × 10^6^–8,28 × 10^6^	−0,051	0,959^a^
		Right Posterior	4,08 × 10^6^ ± 2,36 × 10^6^	1,04 × 10^6^–9,3 × 10^6^	4,21 × 10^6^ ± 2,38 × 10^6^	1,09 × 10^6^–9,89 × 10^6^	−0,566	0,572^a^
		Right Anterior	3,6 × 10^6^ ± 1,37 × 10^6^	1,14 × 10^6^–8,8 × 10^6^	4,2 × 10^6^ ± 1,94 × 10^6^	1,64 × 10^6^–9,81 × 10^6^	−1,203	0,229^a^
	Right Unilateral	Left Anterior	3,31 × 10^6^ ± 2,19 × 10^6^	1,08 × 10^6^–9,99 × 10^6^	4,19 × 10^6^ ± 1,98 × 10^6^	1,29 × 10^6^–9,92 × 10^6^	−2,149	**0,032^a^**
		Left Posterior	2,99 × 10^6^ ± 1,74 × 10^6^	1,05 × 10^6^–9,61 × 10^6^	4,56 × 10^6^ ± 2,47 × 10^6^	1,09 × 10^6^–9,62 × 10^6^	−2,54	**0,011^a^**
		Right Posterior	3,85 × 10^6^ ± 2,64 × 10^6^	1,08 × 10^6^–9,83 × 10^6^	4,57 × 10^6^ ± 2,59 × 10^6^	1,08 × 10^6^–9,9 × 10^6^	−2,746	**0,006** ^a^
		Right Anterior	4 × 10^6^ ± 2,38 × 10^6^	1,13 × 10^6^–9,33 × 10^6^	4,21 × 10^6^ ± 2,04 × 10^6^	1,1 × 10^6^–9,49 × 10^6^	−1,039	0,299^a^
	Bilateral	Left Anterior	3,77 × 10^6^ ± 2,07 × 10^6^	1,11 × 10^6^–8,95 × 10^6^	4,73 × 10^6^ ± 2,46 × 10^6^	1,09 × 10^6^–9,82 × 10^6^	−1,471	0,141^a^
		Left Posterior	3,92 × 10^6^ ± 2,14 × 10^6^	1,15 × 10^6^–9,83 × 10^6^	3,96 × 10^6^ ± 2,67 × 10^6^	1,31 × 10^6^–9,57 × 10^6^	−0,072	0,943^a^
		Right Posterior	4,06 × 10^6^ ± 1,81 × 10^6^	1,47 × 10^6^–8,41 × 10^6^	4,02 × 10^6^ ± 2,24 × 10^6^	1,05 × 10^6^–9,39 × 10^6^	−0,098	0,922^b^
		Right Anterior	4,43 × 10^6^ ± 2,23 × 10^6^	1,39 × 10^6^–9,23 × 10^6^	4,47 × 10^6^ ± 2,49 × 10^6^	1,2 × 10^6^–9,14 × 10^6^	−0,093	0,926^a^
ALPHA	Left Unilateral	Left Anterior	2,88 × 10^6^ ± 1,4 × 10^6^	1,17 × 10^6^–6,83 × 10^6^	3,47 × 10^6^ ± 1,72 × 10^6^	1,7 × 10^6^–9,49 × 10^6^	−1,820	0,069^a^
		Left Posterior	2,57 × 10^6^ ± 1,18 × 10^6^	1,02 × 10^6^–5,56 × 10^6^	3,33 × 10^6^ ± 1,9 × 10^6^	1,12 × 10^6^–9,02 × 10^6^	−2,725	**0,006^a^**
		Right Posterior	4,14 × 10^6^ ± 2,11 × 10^6^	1,03 × 10^6^–7,9 × 10^6^	4,22 × 10^6^ ± 1,64 × 10^6^	1,54 × 10^6^–8,25 × 10^6^	−0,251	0,804^a^
		Right Anterior	2,93 × 10^6^ ± 1,46 × 10^6^	1,01 × 10^6^–6,57 × 10^6^	3,52 × 10^6^ ± 1,97 × 10^6^	1,11 × 10^6^–8,59 × 10^6^	−1,532	0,125^a^
	Right Unilateral	Left Anterior	3,04 × 10^6^ ± 1,87 × 10^6^	1,03 × 10^6^–8,36 × 10^6^	4,1 × 10^6^ ± 2,06 × 10^6^	1,31 × 10^6^–9,59 × 10^6^	−2,437	**0,015^a^**
		Left Posterior	3,21 × 10^6^ ± 1,94 × 10^6^	1,12 × 10^6^–9,11 × 10^6^	4,22 × 10^6^ ± 2,46 × 10^6^	1,06 × 10^6^–9,89 × 10^6^	−2,088	**0,037^a^**
		Right Posterior	3,24 × 10^6^ ± 1,88 × 10^6^	1,06 × 10^6^–8,11 × 10^6^	4,47 × 10^6^ ± 2,41 × 10^6^	1,14 × 10^6^–9,09 × 10^6^	−2,396	**0,017^a^**
		Right Anterior	3,35 × 10^6^ ± 2 × 10^6^	1,05 × 10^6^–9,14 × 10^6^	4,02 × 10^6^ ± 2,25 × 10^6^	1,25 × 10^6^–9,82 × 10^6^	−1,656	0,098^a^
	Bilateral	Left Anterior	3,18 × 10^6^ ± 1,49 × 10^6^	1,04 × 10^6^–7,9 × 10^6^	3,81 × 10^6^ ± 1,69 × 10^6^	1,36 × 10^6^–7,98 × 10^6^	−2,725	**0,006^a^**
		Left Posterior	4,13 × 10^6^ ± 1,98 × 10^6^	1,67 × 10^6^–9,21 × 10^6^	4,11 × 10^6^ ± 2,41 × 10^6^	1,01 × 10^6^–9,26 × 10^6^	−0,010	0,992^a^
		Right Posterior	4,35 × 10^6^ ± 2,24 × 10^6^	1,49 × 10^5^–8,34 × 10^6^	5,36 × 10^6^ ± 2,54 × 10^6^	1,02 × 10^6^–9,94 × 10^6^	−2,165	**0,039^b^**
		Right Anterior	4,56 × 10^6^ ± 1,98 × 10^6^	1,11 × 10^6^–8,32 × 10^6^	4,98 × 10^6^ ± 2,51 × 10^6^	1,22 × 10^6^–9,86 × 10^6^	−1,074	0,291^b^
LOW BETA	Left Unilateral	Left Anterior	3,69 × 10^6^ ± 2,26 × 10^6^	1,28 × 10^6^–9,86 × 10^6^	2 × 10^6^ ± 7,74 × 10^5^	1,21 × 10^6^–4,52 × 10^6^	−4,288	**0,000^a^**
		Left Posterior	3,82 × 10^6^ ± 2,18 × 10^6^	1,16 × 10^6^–9,7 × 10^6^	1,96 × 10^6^ ± 7,47 × 10^5^	1,12 × 10^6^–4,41 × 10^6^	−4,371	**0,000^a^**
		Right Posterior	3,27 × 10^6^ ± 1,75 × 10^6^	1,4 × 10^6^–8,17 × 10^6^	2,63 × 10^6^ ± 1,52 × 10^6^	1,4 × 10^6^–7,94 × 10^6^	−2,602	**0,009^a^**
		Right Anterior	2,72 × 10^6^ ± 1,51 × 10^6^	1,48 × 10^6^–6,55 × 10^6^	2,15 × 10^6^ ± 1,27 × 10^6^	1,05 × 10^6^–6,72 × 10^6^	−3,096	**0,002^a^**
	Right Unilateral	Left Anterior	3,27 × 10^6^ ± 2,41 × 10^6^	1,37 × 10^6^–9,72 × 10^6^	2,63 × 10^6^ ± 1,41 × 10^6^	1,1 × 10^6^–6,02 × 10^6^	−1,553	0,12^a^
		Left Posterior	2,67 × 10^6^ ± 1,53 × 10^6^	1,24 × 10^6^–6,67 × 10^6^	2,33 × 10^6^ ± 1,01 × 10^6^	1,04 × 10^6^–5,39 × 10^6^	−0,812	0,417^a^
		Right Posterior	3,51 × 10^6^ ± 2,01 × 10^6^	1,28 × 10^6^–8,69 × 10^6^	2,73 × 10^6^ ± 1,65 × 10^6^	1,02 × 10^6^–7,01 × 10^6^	−2,540	**0,011^a^**
		Right Anterior	3,33 × 10^6^ ± 2,18 × 10^6^	1,34 × 10^6^–9,85 × 10^6^	2,36 × 10^6^ ± 1,81 × 10^6^	1,04 × 10^6^–8,68 × 10^6^	−2,540	**0,011^a^**
	Bilateral	Left Anterior	3,31 × 10^6^ ± 2,01 × 10^6^	1,27 × 10^6^–7,91 × 10^6^	3,09 × 10^6^ ± 2,02 × 10^6^	1,38 × 10^6^–8,54 × 10^6^	−1,738	0,082^a^
		Left Posterior	3,21 × 10^6^ ± 2,2 × 10^6^	1,27 × 10^6^–9,56 × 10^6^	3,43 × 10^6^ ± 2,27 × 10^6^	1,11 × 10^6^–8,46 × 10^6^	−0,319	0,75^a^
		Right Posterior	2,94 × 10^6^ ± 1,67 × 10^6^	1,17 × 10^6^–8,78 × 10^6^	2,86 × 10^6^ ± 1,93 × 10^6^	1,13 × 10^6^–9,15 × 10^6^	−0,771	0,441^a^
		Right Anterior	3,09 × 10^6^ ± 1,78 × 10^6^	1,46 × 10^6^–8,27 × 10^6^	3,72 × 10^6^ ± 6,71 × 10^6^	1,1 × 10^6^–3,84 × 10^6^	−1,471	0,141^a^
HIGH BETA	Left Unilateral	Left Anterior	3,53 × 10^6^ ± 2,14 × 10^6^	1,33 × 10^6^–9,71 × 10^6^	1,81 × 10^6^ ± 6,39 × 10^5^	1,11 × 10^6^–3,5 × 10^6^	−4,638	**0,000^a^**
		Left Posterior	3,31 × 10^6^ ± 1,92 × 10^6^	1,13 × 10^6^–9,64 × 10^6^	1,78 × 10^6^ ± 7,19 × 10^5^	1,01 × 10^6^–3,9 × 10^6^	−4,782	**0,000^a^**
		Right Posterior	3,06 × 10^6^ ± 1,86 × 10^6^	1,16 × 10^6^–8,76 × 10^6^	2,38 × 10^6^ ± 1,57 × 10^6^	1,11 × 10^6^–7,01 × 10^6^	−3,075	**0,002^a^**
		Right Anterior	3,62 × 10^6^ ± 2,57 × 10^6^	1,25 × 10^6^–9,56 × 10^6^	1,96 × 10^6^ ± 1,45 × 10^6^	1,1 × 10^6^–8,81 × 10^6^	−4,247	**0,000^a^**
	Right Unilateral	Left Anterior	3,35 × 10^6^ ± 2,46 × 10^6^	1,18 × 10^6^–9,37 × 10^6^	2,34 × 10^6^ ± 1,29 × 10^6^	1,1 × 10^6^–5,94 × 10^6^	−2,335	**0,02^a^**
		Left Posterior	2,89 × 10^6^ ± 2,01 × 10^6^	1 × 10^6^–8,88 × 10^6^	2,21 × 10^6^ ± 1,26 × 10^6^	1 × 10^6^–6,85 × 10^6^	−1,944	0,052^a^
		Right Posterior	3,88 × 10^6^ ± 2,94 × 10^6^	1,04 × 10^6^–9,57 × 10^6^	2,67 × 10^6^ ± 1,83 × 10^6^	1,01 × 10^6^–7,74 × 10^6^	−2,417	**0,016^a^**
		Right Anterior	3,47 × 10^6^ ± 2,12 × 10^6^	1,16 × 10^6^–9,47 × 10^6^	2,35 × 10^6^ ± 1,5 × 10^6^	1,12 × 10^6^–7,02 × 10^6^	−2,684	**0,007^a^**
	Bilateral	Left Anterior	2,54 × 10^6^ ± 1,39 × 10^6^	1,02 × 10^6^–5,76 × 10^6^	2,66 × 10^6^ ± 1,65 × 10^6^	1,07 × 10^6^–7,31 × 10^6^	−0,195	0,845^a^
		Left Posterior	3,89 × 10^6^ ± 2,45 × 10^6^	1,23 × 10^6^–9,12 × 10^6^	2,81 × 10^6^ ± 1,87 × 10^6^	1,17 × 10^6^–8,11 × 10^6^	−2,273	**0,023^a^**
		Right Posterior	2,81 × 10^6^ ± 1,77 × 10^6^	1,34 × 10^6^–7,95 × 10^6^	2,39 × 10^6^ ± 1,47 × 10^6^	1,21 × 10^6^–7,17 × 10^6^	−1,347	0,178^a^
		Right Anterior	2,76 × 10^6^ ± 1,76 × 10^6^	1,23 × 10^6^–8,04 × 10^6^	2,07 × 10^6^ ± 1,11 × 10^6^	1,01 × 10^6^–5,9 × 10^6^	−2,232	**0,026^a^**
GAMMA	Left Unilateral	Left Anterior	3,27 × 10^6^ ± 2,33 × 10^6^	1,04 × 10^6^–9,14 × 10^6^	1,84 × 10^6^ ± 9,99 × 10^5^	1,09 × 10^6^–5,31 × 10^6^	−4,083	**0,000^a^**
		Left Posterior	3,08 × 10^6^ ± 2,66 × 10^6^	1,11 × 10^6^–9,89 × 10^6^	2,22 × 10^6^ ± 1,86 × 10^6^	1,03 × 10^6^–9,84 × 10^6^	−3,569	**0,000^a^**
		Right Posterior	3,2 × 10^6^ ± 2,21 × 10^6^	1,06 × 10^6^–8,87 × 10^6^	2,11 × 10^6^ ± 1,28 × 10^6^	1,04 × 10^6^—6 × 10^6^	−3,733	**0,000^a^**
		Right Anterior	2,74 × 10^6^ ± 2,11 × 10^6^	1,01 × 10^6^–9,73 × 10^6^	1,76 × 10^6^ ± 1,13 × 10^6^	7,65 × 10^5^–6,77 × 10^6^	−4,309	**0,000^a^**
	Right Unilateral	Left Anterior	2,92 × 10^6^ ± 2,13 × 10^6^	1,07 × 10^6^–9,8 × 10^6^	1,97 × 10^6^ ± 1,24 × 10^6^	1,11 × 10^6^–7,17 × 10^6^	−2,910	**0,004^a^**
		Left Posterior	2,93 × 10^6^ ± 2,4 × 10^6^	1,02 × 10^6^–9,67 × 10^6^	2,55 × 10^6^ ± 3,09 × 10^6^	1,08 × 10^6^–1,77 × 10^6^	−1,964	**0,049^a^**
		Right Posterior	3,33 × 10^6^ ± 2,56 × 10^6^	1,22 × 10^6^–9,32 × 10^6^	1,94 × 10^6^ ± 1,17 × 10^6^	1,01 × 10^6^–6,12 × 10^6^	−3,240	**0,001^a^**
		Right Anterior	3,32 × 10^6^ ± 2,48 × 10^6^	1,2 × 10^6^–9,22 × 10^6^	2,47 × 10^6^ ± 1,71 × 10^6^	1,17 × 10^6^–9,41 × 10^6^	−2,376	**0,018^a^**
	Bilateral	Left Anterior	3,44 × 10^6^ ± 2,3 × 10^6^	1,07 × 10^6^–9,13 × 10^6^	2,06 × 10^6^ ± 1,27 × 10^6^	1,09 × 10^6^–6,24 × 10^6^	−2,766	**0,006^a^**
		Left Posterior	3,39 × 10^6^ ± 2,38 × 10^6^	1,15 × 10^6^–9,82 × 10^6^	2,81 × 10^6^ ± 1,88 × 10^6^	1,07 × 10^6^–7,47 × 10^6^	−1,512	0,131^a^
		Right Posterior	3,42 × 10^6^ ± 2,61 × 10^6^	1,25 × 10^6^–9,77 × 10^6^	2,13 × 10^6^ ± 1,08 × 10^6^	1,12 × 10^6^–4,92 × 10^6^	−2,211	**0,027^a^**
		Right Anterior	3,02 × 10^6^ ± 2,26 × 10^6^	1,05 × 10^6^–9,22 × 10^6^	2,03 × 10^6^ ± 9,45 × 10^5^	1,11 × 10^6^–4,9 × 10^6^	−2,499	**0,012^a^**

^a^ Wilcoxan Sign Rank Test.

^b^ Paired-Sample *t*-Test.

The bold values are *p* < 0.05.

## 4 Discussion

Auricular VNS is a safe and well-tolerated intervention with intriguing physiological and behavioral effects in recent years (Redgrave et al., 2018). Despite the wide variety of potential therapeutic applications, there is still debate about the basis of auricular VNS. The effects of a single session of auricular VNS application on cerebral and peripheral activity in healthy individuals were evaluated with pre- and post-stimulation EEG and EMG measurements in randomized unilateral left, unilateral right and bilateral stimulation groups. The results obtained in this study were interesting. The findings of our study show that a single session of auricular VNS increases the muscle activation of the wrist extensor muscle group on the opposite side of the ear where stimulation is performed, but has no effect on muscle strength.

In the literature, there is a study in which a similar method is used to measure the hand grip strength simultaneously while evaluating the activation of the muscles in the forearm with EMG (Kinali and Üçsular, 2018). On the other hand, no study examining the effect of auricular VNS on muscle activation has been found. The findings of our study suggest that auricular VNS can also be included in rehabilitation programs designed for protective, preventive and therapeutic purposes related to physical exposures that cause work-related musculoskeletal diseases due to its contralateral effect on muscle activation. In addition, due to the contralateral effect of the auricular VNS on muscle activation, stimulation from the side of the central injury may be more effective in stroke patients.

It has been reported in the literature that stimulation of the left or right unilateral auricular VNS is not expected to produce different physiological effects because afferent information from both sides is centrally combined in the brainstem. However, it is noted that simultaneous activation by left and right auricular VNS can potentially enhance stimulation effects due to increased sensory input in the brainstem (Kaniusas et al., 2019). The auricular VNS probably affects the CNS origin of the corticospinal tracts ipsilaterally. It is also reported that auricular VNS reduces increased muscle tone (Kampusch et al., 2015). The fact that the findings we obtained in our study are in the opposite direction can be explained by possible regulatory effect of the auricular VNS (increasing decreased activity and decreasing increased activity).

Although the literature generally tells us that there is no difference in effect and side effects of right or left auricular VNS, corticospinal pathway or muscle activation may be affected differently by stimulation (Kim et al., 2022). There is limited evidence on differential side stimulation effects and further studies are needed to confirm this. In one study, it was reported that right and left-sided auricular VNS applied in healthy subjects, similar to our study, may affect heart rate variability (HRV) parameters differently (Machetanz et al., 2021b). Chen et al. (2023) hypothesized that left auricular VNS can increase cortical excitation in left brain regions and accelerate right hand reaction. It was found that there was a significantly greater decrease in right hand reaction time in the auricular VNS group than in the control group. However, no such difference could be detected in left hand reaction time. In summary, the brain does not have a fully symmetrical structure in terms of functionality. Therefore, different effects are always possible with right and left auricular VNS.

Ünal et al. (2022), investigated the changes that may occur in the hand grip strength of the cervical myofascial pain syndrome patients with the 30 min of auricular VNS applied in addition to the ischemic compression application and stretching exercises. The control group was given only ischemic compression and stretching exercises. Participants in both groups received a total of 10 sessions of treatment, 5 days a week. When the changes observed in the VNS and control groups at the end were compared, the changes in Jamar dynamometer (hand dynamometer that measures hand grip strength) scores in the VNS group were found to be statistically significantly different compared to the control group. In our study, no significant difference was found in hand grip strength with a single session auricular VNS that we applied in all three groups filled with healthy people. A single session of stimulation may not be sufficient to produce a significant increase in muscle strength. A single session of auricular VNS probably increases muscle activity, but this activity is not sufficient to increase muscle strength. It may be necessary to perform more sessions of auricular VNS sessions to see if it improves muscle strength.

Sclocco et al. (2019) evaluated the response of the brainstem with functional MRI to the application of auricular VNS during inhalation and exhalation. It has been reported that the auricular VNS applied during exhalation increases the stimulation effect reaching the NTS, and also improves cardiovascular modulation. The results we obtained with EMG measurements suggest that the response in the brain stem may be related to the application of auricular VNS.

It has also been described in previous studies that there is a shift in autonomic functions towards parasympathetic dominance with the increase in HRV during auricular VNS (Clancy et al., 2014; De Couck et al., 2017). Although the vagal effect on the ANS has been proven, there are still unanswered questions regarding the target of auricular stimulation, its effect on cortical brain areas, and the underlying anatomical and physiological mechanisms of the interaction between the ANS and the CNS (Silvani et al., 2016; Cakmak, 2019; Kaniusas et al., 2019). Research on auricular VNS in the literature is focused on clinical trials showing potential therapeutic benefits. However, the neurophysiological effects of this stimulus on brain activity are still unclear (Gianlorenco et al., 2022). In the literature, we have not come across a study comparing the effects of right, left and bilateral auricular vagus nerve stimulation in healthy persons so far.

In a study by Machetanz et al. (2021a) to evaluate changes corresponding to HRV and cortical activation patterns during auricular VNS, EEG and electrocardiography (ECG) measurements were performed simultaneously with stimulation in 13 healthy participants. Stimulation was made in the ear from two different locations: the inner tragus/cymba concha and the outer targus/crus helicis. It has been reported that the increase in theta band power in the frontal region was the most significant change corresponding to the increase in HRV in stimulation from both positions. However, it has been determined that inner tragus/cymba concha stimulation causes limited alpha and beta band power increases and there is a significant decrease in fronto-parietal gamma band power. We have seen that the concomitant tragus and concha stimulation (left, right or both) is effective to induce changes in the brain and peripheral muscles.

In another study by Ricci et al. (2020), male participants were included between the ages of 25–45 years, and all participants received both real and placebo transcutaneous VNS administration for 60 min. In the real stimulation application, the external acoustic meatus located in the inner side of the left ear tragus was stimulated, and in the placebo application, the left earlobe was stimulated. EEG measurements before and after real stimulation revealed an increase in power spectrum activity in the delta frequency band, while placebo stimulation had no effect. In our study, an increase in theta band power was detected in the left anterior region in the left auricular VNS group, and in the left anterior, left posterior and right posterior regions in the right auricular VNS group. The results of our study in the left-unilateral and right-unilateral auricular VNS groups are similarly consistent with studies showing that tragus and concha regions stimulation causes delta/theta increase in the frontal region (Gianlorenco et al., 2022).

There are studies in the literature showing that the frontal cortex is a cortical area that supports parasympathetic activity and that there is a neurocardiac connection between HRV parameters and the activity of low-frequency (delta/theta) brain waves (Thayer et al., 2012).

In a study by Patron et al. (2019), ECG and EEG measurements were recorded for 5 min during rest in 38 healthy individuals. To evaluate the effect of the relationship between heart-beats and cortical control on vagal activity, the interval between the longest beat and the shortest beat for each participant was determined. It has been reported that the decrease in delta power in the prefrontal and frontocentral areas before the R-wave in the ECG signal increases the likelihood of a longer pulse interval compared to the shorter pulse interval. The view that phasic activation/deactivation of prefrontal areas is a cardiac brake of the frontal cortex by modulating vagal control of resting heart rate has been supported. In another study, “neurocardiac connectivity score” indexed by the relationship between delta power and heartbeat length was used, taking into account the results obtained by Patron et al. (2019). In this study by Keute et al. (2021), the cardiac effects of auricular VNS were evaluated in 44 healthy adults and it was reported that auricular VNS showed parasympathetic activation. Similarly, in our study, EEG results obtained in the direction of an increase in the band power of relatively lower-medium frequency brain waves (theta, alpha) and a decrease in the band power of higher frequency brain waves (beta, gamma) in different regions of the brain, after auricular VNS. It can be explained by the fact that auricular VNS application creates parasympathetic activation. We did not measure autonomic nervous system activity, either parasympathetic nor sympathetic, but band power changes were in favor of increased parasympathetic activity.

It has been reported that increased sympathetic activity exacerbates spontaneous pain at the trigger points and ANS dysfunctions are related to chronic musculoskeletal pain (Ge et al., 2006). Although the etiology of Fibromyalgia Syndrome (FMS) has not been fully elucidated, it is thought that ANS dysfunction may also play a role in its causes. In a study conducted by Kutlu et al. (2020) on female patients with FMS, the effect of auricular VNS applied in addition to exercise therapy on pain and quality of life was evaluated. In this study, which included a total of 60 participants, there were two groups of 30 participants each. Only exercise therapy was applied to the control group, and bilateral auricular VNS was applied to the study group in addition to exercise therapy. Participants in both groups received 20 sessions of treatment. According to the results of the measurements obtained after the treatment, it was observed that there was an improvement in pain, depression, anxiety, functionality, and quality of life scores in both groups compared to before treatment. However, it was determined that the scores were higher in the auricular VNS group, although it was not statistically significant. We have seen that the auricular VNS can affect muscle activity and we think that the pain centers in the brain may also be affected in parallel with the changes in the EEG.

In another study by Yüksel et al. (2019), the therapeutic effects of acupuncture and transcutaneous electrical nerve stimulation (TENS) applications in patients with FMS were examined through quantitative EEG changes. It has been reported that delta/theta band power on the frontal region and theta band power on the right posterior region of individuals with FMS before treatment were lower than those of healthy controls. An increase in the alpha power of the left anterior region and a decrease in pain scores were detected in the FMS group treated with TENS. In the acupuncture group, an increase in the alpha power of the right posterior and left posterior regions and a decrease in the pain score were detected after the treatment. It has been determined that there is a decrease in pain and an increase in inhibitory activity after TENS and acupuncture applications in patients with FMS. Similar to the results obtained after acupuncture and TENS application in our research, VNS performed from different ears caused an increase in alpha power in different parts of the brain. In our study, alpha power was increased in the left posterior region in the left-unilateral auricular VNS group, in the left anterior, left posterior and right posterior regions in the right-unilateral auricular VNS group, and in the left anterior and right posterior regions in the bilateral auricular VNS group.

The alpha waves of EEG reflect the activity of inhibitory neurons. Decreased alpha band power indicates dysfunction in the thalamocortical circuit, where inhibition of trivial information cannot be achieved (Klimesch et al., 2007). Therefore, our results are consistent with studies supporting the idea that auricular VNS can be used as an alternative approach in the treatment of chronic pain for patients with FMS (Martins et al., 2021; Molero- Chamizo et al., 2022).

A study examined the acute and chronic effects of invasive stimulation of the left cervical vagal nerve on brain activity in nine individuals with Crohn’s Disease. While spectral power increases were observed in delta and theta bands in the frontal, temporal and occipital regions with acute stimulation, alpha power was decreased during the clinical recovery of patients after 12 months of chronic VNS (Kibleur et al., 2018). In our study, the acute effect of stimulation on the EEG results was similarly in the direction of an increase in theta band power. However, it has also been described in epilepsy and depression, where clinical effects of VNS are expected after at least six months of treatment (Conway et al., 2013; Kibleur et al., 2018; Hamer and Bauer, 2019). The effect of VNS on the CNS explains the gradual and progressive slow cortical remodeling.

Considering the EEG results we obtained, auricular VNS can cause activity changes in both the ipsilateral and contralateral hemispheres. However, EMG results indicate that the effect of ipsilateral stimulation may be localized to one-half of the body in the periphery. Similarly, auricular VNS applied from the right or left side may have different specific effects on visceral organs. According to the EEG results, we can state that the auricular stimulation from the right-unilateral has a more global effect on the brain than the stimulation made from the left-unilateral. Right-unilateral auricular stimulation may be preferred especially in neurological and psychiatric disorders involving the CNS if bilateral stimulation is somewhat contraindicated.

Various side effects, including local skin irritation, headache, and nasopharyngitis associated with auricular VNS, have been reported in the literature (Ben-Menachem et al., 2015; Redgrave et al., 2018). None of the participants included in our study experienced such side effects. As a result, changes to be detected as a result of EEG and EMG measurements performed before and after stimulation from different ears in healthy individuals may give an idea about the use of auricular VNS in patients. Larger scale, randomized, prospective studies are needed to evaluate how long the altered EMG and EEG activity lasts after VNS applications in different ears and to confirm the findings.

## 5 Limitation

The Emotiv EPOC + EEG device was not measuring the delta frequency. Therefore, the average band power of the delta frequency could not be calculated. In addition, the control group (non-stimulated group) was not used in this study. On the other hand, how long the EEG activity, which changed with VNS applications from different ears, lasted could not be evaluated. Intermittent measurements made after VNS are needed to verify this results. Results could be checked by repeating the same protocol on the same participants at another time. The lack of measurement of autonomic nervous system activity can also be noted as a limitation.

## 6 Conclusion

In our study, it was observed that the central and peripheral effects of auricular VNS applied from different ears may be different. Future studies are needed to compare the effects of similarly applied stimulation from different ears in different disease groups. In this way, more specific and more effective use of auricular VNS can be achieved for each disease and patient. Our findings suggested that the projection of the auricular VNS in the CNS may also affect the corticospinal tracts. In the future, there is a need for large-scale studies in accordance with the criteria of evidence-based medicine, in which the effects of different auricle stimulations and separate stimulation parameters are investigated using many imaging methods in different clinical situations.

## Data Availability

The raw data supporting the conclusion of this article will be made available by the authors, without undue reservation.
